# Recovery From Long COVID: The Role of Bioelectric Meridian Therapy in Restoring Health and Well-Being

**DOI:** 10.7759/cureus.76279

**Published:** 2024-12-23

**Authors:** Praveen Mallari, Tracy Taulier, Mohammad A Kamal

**Affiliations:** 1 Zoology, Indira Gandhi National Tribal University, Amarkantak, IND; 2 Bioelectric Meridian Therapy, Academy of Bioelectric Meridian Massage Australia, Brisbane, AUS; 3 Biochemistry, Princess Nourah Bint Abdul Rahman University, Riyadh, SAU

**Keywords:** bioelectric meridian therapy, complementary therapy, covid-19 recovery, loss of taste and smell, mental fatigue, post-covid syndrome

## Abstract

This case report explores the journey to a healthier life of a 57-year-old man who stayed athletic after contracting COVID-19 during a trip to a foreign country. He had minimal symptoms in the beginning. He started with a dull cough, but the symptoms then progressed to loss of taste and smell, mental fatigue, and nerve problems. In this case study, traditional cures helped only to some extent. Out of frustration with these symptoms, the patient started bioelectric meridian therapy (BMT) in August 2021 and benefited from it. The patient described progressive sensation, cognition, and muscle power enhancement during six months of BMT sessions. The therapy relieved the muscles' stiffness and regulated the bio-energy circulation to improve the general quality of life and ease physical exertion operations. The case affirms how supportive modalities such as BMT help handle the after-effects of COVID-19. The patients' recovery involved precise mental concentration, return of appetite, and regained vigor, which improved the patient's life. This case report shows how integrative approaches may be helpful when it comes to the reactivation of health processes after virus infection.

## Introduction

Bioelectric meridian therapy (BMT) uses electrical currents to stimulate specific meridian points on the body [1]. The concept originates from traditional Chinese medicine, which posits that health is influenced by the free flow of bioelectrical energy through channels called meridians. However, this concept is not universally accepted in scientific communities [2]. Bioelectric therapies are believed to aid in managing symptoms of many chronic diseases and pain conditions, individually or in combination with other treatments [3]. In some conditions, it can assist in the control of asthma, hypertension, migraines, arthritis, gastrointestinal disorders, depression, anxiety, insomnia, and muscle aches [4].

The electrical stimulation utilized is slight and enhances the flow of energy and blood, reduces inflammation, releases stereotyped endorphins, changes the signals of specific nerves, and produces other physiological altering responses contingent on the positions of electrodes [5]. It can manage symptoms but does not directly address disease states. TENS units and microcurrent therapy devices are bioelectric devices used on the skin at specific sites. Acute problems typically take 30 minutes, while chronic concerns take one to two hours per session, one to two times per week. The effect is temporary, and the treatments typically require repetition [6]. BMTs have been the subject of limited research, and most of the findings have been from minor pilot investigations. This also means that more significant trials are still needed to confirm the efficacy of many diseases. Complementary therapies, including BMT, may alleviate symptoms. However, their effects on underlying disease mechanisms require further investigation [7]. If employed optimally, it tends to go well with other lifestyle modifications to promote health. However, progress is still needed in terms of stronger clinical endorsement.

Some earlier research, including needle approaches to alter bioelectric signals within the body, has been identified to offer anti-inflammatory and immunomodulating effects [8]. Those preaching bioelectric therapies posit that electroacupuncture can complement overall well-being, leading to better results. Nonetheless, no specific proof has been presented supporting such therapies as improving COVID-19 symptoms or helping patients recover. Randomized, controlled trials that compare bioelectric treatments with standard medical practices in COVID-19 cases have not been conducted at an enlarged measure or with necessary rigor. However, there is a wealth of information in the literature on COVID-19 research, including numerous publications by Kamal and his global team [9-24].

## Case presentation

Patient information, infection, and symptoms

The patient became a victim of COVID-19 upon his return to Australia after working in the United States. He then developed other COVID-19 symptoms, such as fatigue, dry cough, and a loss of taste and smell. He attributed these symptoms to the air conditioning in his hotel quarantine. By day 12, he got tested on October 10, 2020; the results confirmed he had contracted COVID-19 (COVID-19 test positive). By October 30, 2020, after completing his quarantine, he experienced neurological symptoms such as numbness and cognitive issues, including forgetfulness. He also stated that, due to poor pulmonary and muscular performances and an exhausted and anxious mental state, he felt less energetic and physically weaker after the illness. These challenges were aggravated by a lack of focus and concentration in executing tasks and making decisions.

Physical weakness and adaptation after illness

After trying to return to his everyday life, he realized his muscle strength had reduced, particularly during the most straightforward exercises. He experienced reduced pulmonary function, leading to decreased endurance, which was particularly noticeable when driving long distances. This decreased his endurance, which was evident when he took a long drive. These physical limitations, which he blamed on COVID-19, meant that the illness slowly affected his health physically, and the effects only manifested when he returned to his routine activities.

Psychological and nutritional recovery efforts

Coping with tiredness of the brain and stress due to the illness, he consulted a doctor. His doctor considered that he might be tired due to recent changes and prescribed vitamins, followed by counseling (October 30, 2020), which did not help. A naturopath later noticed that there was a deficiency possibly in his mental health and recommended more zinc. Concerning the changes in the diet, the amount of fruits, vegetables, and meat seemed to assist in physical healing. However, as for the head, he noted that it might take quite a while for his mental state to regain its normal state. Fed up with the regular treatments, he sought BMT because the standard treatments were not helpful to him.

Healing BMT

**Figure 1:**
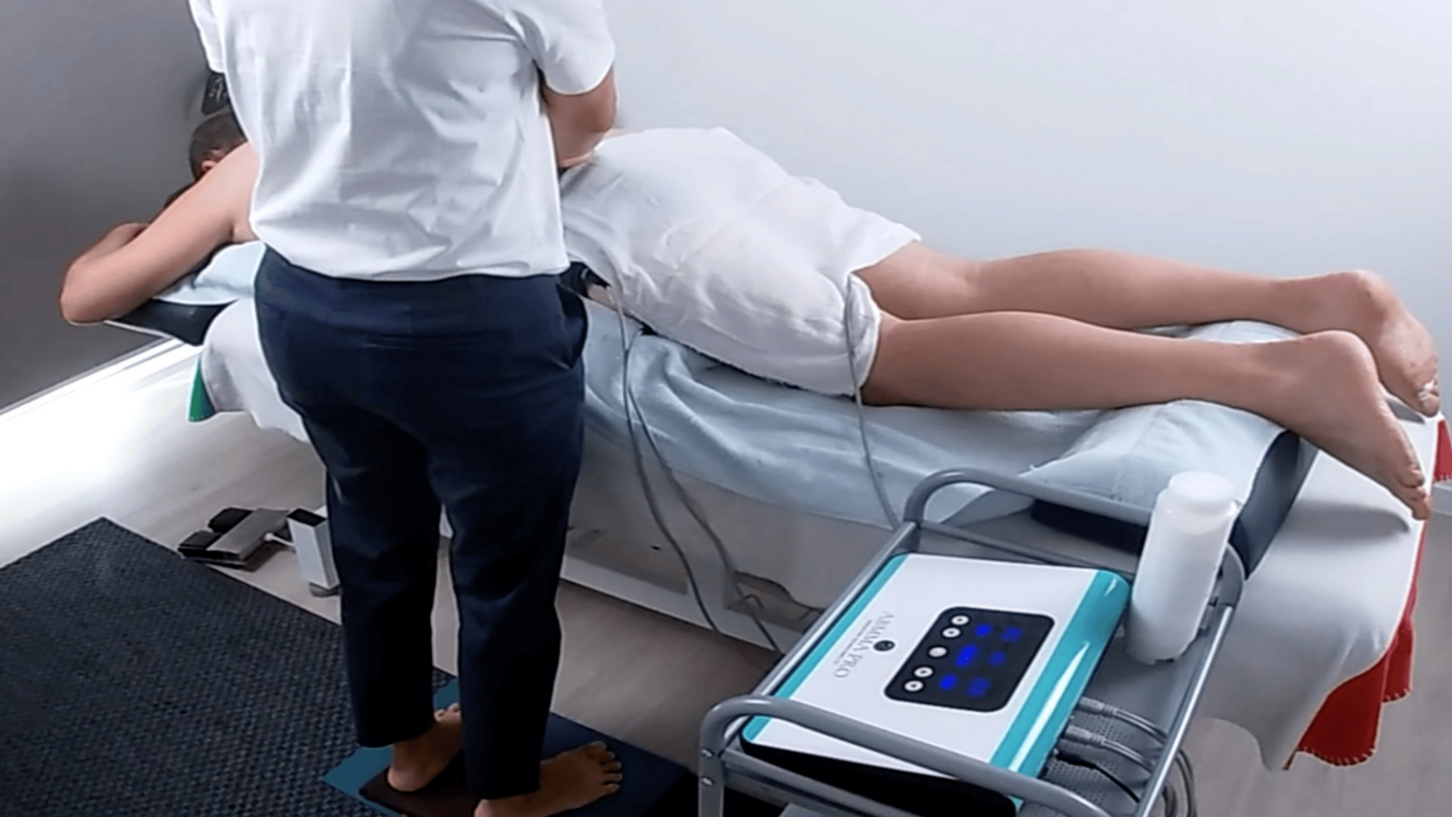
BMT practicing overview to create a concept for the practical session: The purpose of this image is to provide a concise overview of the BMT therapy session, which clearly illustrates the patient’s prone position on the massage table, the BMT device on the side trolley, and the visibility of the pad cables. BMT: Bioelectric Meridian Therapy.

The principal of the Academy of Bioelectric Meridian Massage Australia (ABMMA) shared his experience that using BMT to recover after COVID-19 severely impacted his health. He turned to doctors, naturopaths, and counselors for months before going for BMT in search of permanent relief for standard symptoms, including depression, loss of taste and smell, and fatigue. Feeling hopeless, he decided to go for BMT (Figure 1). The therapist discovered obstruction locations in the first session, indicating that BMT unblocks channels to revive energy (August 23, 2021). After BMT started, he came to his senses, felt hungry, and regained strength, enabling him to go swimming and cycling.

The BMT sessions made him feel less closed down, and his perceptions and spirits improved after more than six months of BMT treatment. The sessions helped him to get his taste and smell back to nearly 80% of their normal state. The therapy also positively decreased the individual's muscle tightness and spasm, particularly in the jaw and spine muscles, thus improving the blood and oxygen supply to the affected areas. Over a year into the process​​​​, he noted ​​​the differing health benefits of BMT, including relief ​​​​​from the physical and psychological symptoms. He has also noticed a gradual improvement in all his senses, mental functions, and physical health with frequent BMT sessions.

The patient admitted he felt hope and slowly observed significant positive changes in his health after beginning BMT sessions. Subsequently, within six months of testing positive for COVID-19, he started feeling some form of skin sensitization and even regained the ability to taste and smell. He began to think and function as before, which changed his perception of situations and improved his brain activity. After six months of BMT, he reported no symptoms, pain, or discomfort. He opined that BMT helped him regain vital aspects of his health and well-being.

## Discussion

The current case describes how, as an augmentation to conventional biomedical protocols, BMT can be employed in eradicating viral symptoms such as COVID-19 sensory disorder and mental health problems, as well as the contribution of complementary therapies across the entire patient journey.

The patient was symptomatic and continued to suffer from sensory dysfunction even after the acute stage of COVID-19 had resolved. Six months after the onset of COVID-19 symptoms, the patient reported a markedly reduced quality of life. After beginning BMT, the patient noted only gradual sensory improvement, indicating that more research on treatments beyond traditional medicine should occur post-COVID-19 recovery [25]. This study presents a rare case of viral infection complicated by a sensory deficit extending beyond the viral isolation period. The patient also seemed to attain some degree of sensory function through BMT, which points to an inherent guarantee governing a factor of bioelectric energy flow and body blockages that specific therapies could remedy [26]. Studies should conduct more research in these areas to ascertain if BMT can help as a complementary treatment for people with chronic sensory impairment.

For this reason, the patient's experience reveals that post-COVID mental health difficulties defy the conventional medical approach. It calls for other treatments compatible with new research, showing that therapies addressing the psychological and physical self may benefit people with long COVID-19 [27]. Positive psychological changes observed after BMT suggest that it can improve mental health and emotional stability [28].

The patient's case suggests other treatment solutions, such as BMT, can help recover from long-term COVID-19. At first, the patient was skeptical but reported that BMT was helpful in some cases where other approaches were not [29]. After the first session, he felt subjectively better psychologically and more optimistic, and over six months, he regained 80% of his taste and smell. Physical improvements included increased energy, relaxed muscles, and reduced pain [30].

As BMT is employed to unblock the ion channels and enhance energy flow, it can manage other biological symptoms such as post-viral fatigue and dysesthesia. Thus, it has shown the use of holistic therapies for restoring strength and endurance in post-illness patients and the possibility of swimming and biking again. Patients frustrated with conventional medication embrace integrated approaches, combining modern and complementary medicines. BMT improved the patient’s symptoms, such as restoring 80% of taste and smell over six months and reducing pain and fatigue.

This case is a clear example of how patient-centered care needs to include attention to patients' physical and mental health. Thus, the response to BMT is that energy medicine should be the vital healing approach to long-term COVID-19, pointing at the necessity of further exploring complementary treatments as part of the chronic care model for COVID-19 patients.

## Conclusions

Hence, the restoration of patients from the impact caused by COVID-19 indicates that the effects on physical, mental, and near-sensory systems may be slow and varying. In the beginning, even when he sought conventional therapeutic approaches, he could not find much comfort. His symptoms included loss of taste and smell, mental confusion, and a tired feeling, which partially resolved after starting BMT. The BMT enhanced his sense of taste and smell, thinking processes, and energy levels. For the first time, he received something other than just a pill for "the real thing," meaning dealing with energy flow interferences to manage his physical and psychological issues. It not only restored comfort and reduced discomfort during the day but also renewed enthusiasm for the patient's routine. This case report shows the effectiveness of traditional Amity, such as BMT, in recovery programs depending on the late effects of COVID-19 outcomes.
